# Supplementary material for: Hospitalizations and Clinical Outcome in Metastatic Colorectal Cancer During Regorafenib or TAS-102 Therapy
Source: Cancers (Basel). 2020 Sep 30;12(10):2812. doi: 10.3390/cancers12102812 (PMC7599669; doi:10.3390/cancers12102812)
Supplement: Supplementary file 1 [file cancers-12-02812-s001.pdf]

Supplementary Materials

Hospitalizations and Clinical Outcome in Metastatic Colorectal Cancer During Regorafenib or TAS-102 Therapy

Florian Huemer, Gudrun Piringer, Verena Schlintl, Hubert Hackl, Gabriel Rinnerthaler, Josef Thaler, Richard Greil and Lukas Weiss

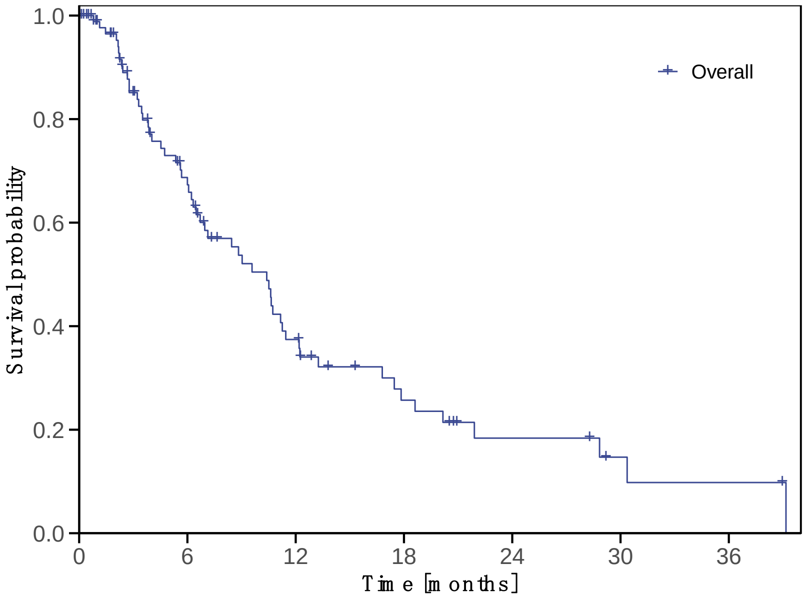

Figure S1. Overall survival from initiation of third-line therapy with regorafenib or TAS-102 in 93 mCRC patients. The tick marks on the curves represent censored patients.

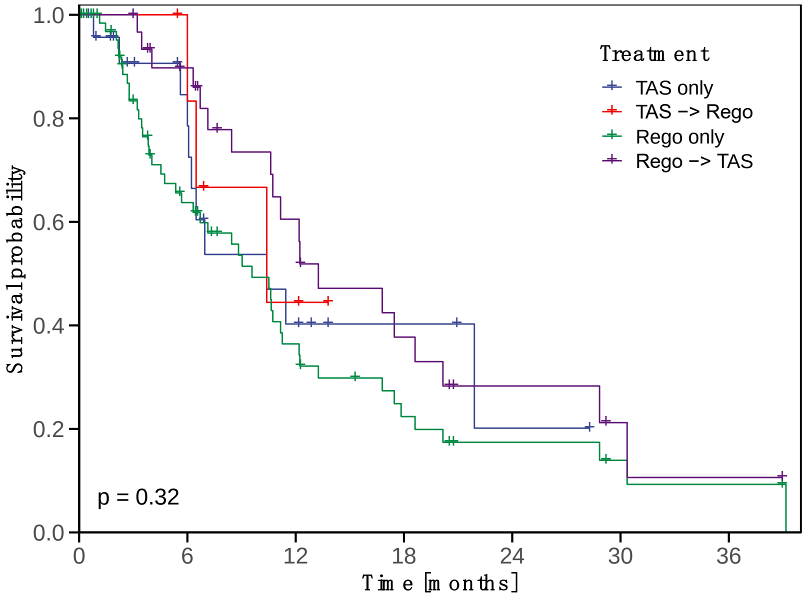

Figure S2. Overall survival from initiation of third-line therapy with regorafenib or TAS-102 according to therapy sequence in 93 mCRC patients. Adjusted survival curves using Cox proportional hazards model. Cross-over was taken into account as a time-dependent covariate beginning with the

start of fourth line treatment in order to avoid immortal time bias. The tick marks on the curves represent censored patients.

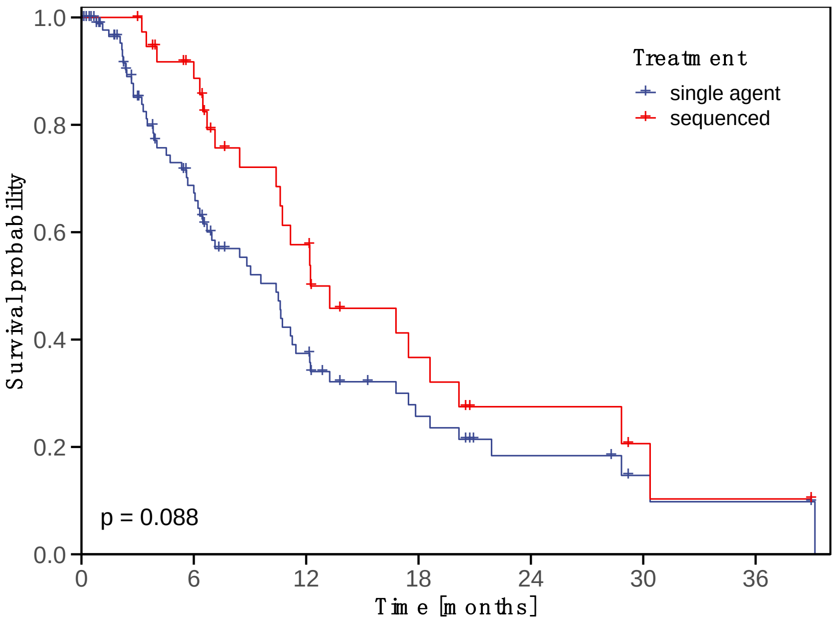

Figure S3. Overall survival from initiation of third-line therapy with regorafenib or TAS-102 according to single agent therapy or sequenced therapy. Adjusted survival curves using Cox proportional hazards model. Cross-over was taken into account as a time-dependent covariate beginning with the start of fourth-line treatment in order to avoid immortal time bias. The tick marks on the curves represent censored patients.

Table S1. Comparison of baseline characteristics between hospitalized and never-hospitalized mCRC patients during third-line or third- and fourth-line therapy with regorafenib or TAS-102.

**Parameter**		**3rd Line Therapy Only with Regorafenib or TAS-102 (*n* = 55)**		
		**Never-Hospitalized** ***n*** **= 26 (%)**	**Hospitalized ≥ 1** ***n*** **= 29 (%)**	***p*-Value**
Age at 3rd line start	< 65 years<br>≥ 65 years	11 (42)<br>15 (58)	13 (45)<br>16 (55)	0.85
Sex	male<br>female	16 (62)<br>10 (38)	15 (52)<br>14 (48)	0.46
Primary tumor status	resected<br>not resected	20 (77)<br>6 (23)	26 (90)<br>3 (10)	0.20
Detection of metastases	synchronous<br>metachronous	15 (58)<br>11 (42)	16 (55)<br>13 (45)	0.85
Sidedness	left<br>right	20 (77)<br>6 (23)	19 (66)<br>10 (34)	0.35
Ascites at 3rd line start	no<br>yes	23 (89)<br>3 (11)	26 (90)<br>3 (10)	0.89
RAS status	wild-type<br>mutant	10 (38)<br>16 (62)	18 (62)<br>11 (38)	0.08

BRAF status	wild-type<br>mutant<br>NA	17 (94)<br>1 (6)<br>8	22 (100)<br>0 (0)<br>7	0.26
Microsatellite status	MSS<br>MSI<br>NA	14 (100)<br>0 (0)<br>12	17 (94)<br>1 (6)<br>11	0.37
3rd line therapy	regorafenib<br>TAS-102	17 (65)<br>9 (35)	21 (72)<br>8 (28)	0.57
ECOG PS at 3rd line start	ECOG PS 0-1<br>ECOG PS 2-3<br>NA	14 (82)<br>3 (18)<br>9	13 (57)<br>10 (43)<br>6	0.09
Sarcopenia at 3rd line start	yes<br>no<br>NA	7 (64)<br>4 (36)<br>15	6 (50)<br>6 (50)<br>17	0.51

**Parameter**		**3rd and 4th Line Therapy with Regorafenib and TAS-102 (or Vice Versa) *n* = 38**		
		**Never-Hospitalized *n* = 24 (%)**	**Hospitalized ≥ 1 *n* = 14 (%)**	***p*-Value**
Age at 3rd line start	< 65 years<br>≥ 65 years	11 (46)<br>13 (54)	8 (57)<br>6 (43)	0.50
Sex	male<br>female	16 (67)<br>8 (33)	7 (50)<br>7 (50)	0.31
Primary tumor status	resected<br>not resected	18 (75)<br>6 (25)	11 (79)<br>3 (21)	0.80
Detection of metastases	synchronous<br>metachronous	19 (79)<br>5 (21)	12 (86)<br>2 (14)	0.62
Sidedness	left<br>right	18 (75)<br>6 (25)	10 (71)<br>4 (29)	0.81
Ascites at 3rd line start	no<br>yes	23 (96)<br>1 (4)	13 (93)<br>1 (7)	0.69
RAS status	wild-type<br>mutant	12 (50)<br>12 (50)	6 (43)<br>8 (57)	0.67
BRAF status	wild-type<br>mutant<br>NA	19 (100)<br>0 (0)<br>5	10 (100)<br>0 (0)<br>4	NA
Microsatellite status	MSS<br>MSI<br>NA	20 (100)<br>0 (0)<br>4	7 (88)<br>1 (12)<br>6	0.11
3rd line therapy	regorafenib<br>TAS-102	19 (79)<br>5 (21)	12 (86)<br>2 (14)	0.62
ECOG PS at 3rd line start	ECOG PS 0-1<br>ECOG PS 2-3<br>NA	18 (100)<br>0 (0)<br>6	14 (100)<br>0 (0)<br>0	NA

	yes	3 (27)	6 (67)	
Sarcopenia at 3rd line start	no	8 (73)	3 (33)	0.08
	NA	13	5	

ECOG PS: Eastern Cooperative Oncology Group performance status, NA: not available, MSI: microsatellite instability, MSS: microsatellite stability.

© 2020 by the authors. Licensee MDPI, Basel, Switzerland. This article is an open access article distributed under the terms and conditions of the Creative Commons Attribution (CC BY) license (http://creativecommons.org/licenses/by/4.0/).
